# Reduced expression of phosphorylated ataxia-telangiectasia mutated gene is related to poor prognosis and gemcitabine chemoresistance in pancreatic cancer

**DOI:** 10.1186/s12885-023-11294-3

**Published:** 2023-09-06

**Authors:** Jingyu Xun, Hideo Ohtsuka, Katsuya Hirose, Daisuke Douchi, Shun Nakayama, Masaharu Ishida, Takayuki Miura, Kyohei Ariake, Masamichi Mizuma, Kei Nakagawa, Takanori Morikawa, Toru Furukawa, Michiaki Unno

**Affiliations:** 1 Department of Surgery, Tohoku University Graduate School of Medicine, 1-1 Seiryo-machi, Aoba-ku, Sendai, 980-8574 Miyagi Japan; 2 Department of Investigative Pathology, Tohoku University Graduate School of Medicine, 1-1 Seiryo-machi, Aoba-ku, Sendai, Miyagi Japan

**Keywords:** Apoptosis, Ataxia telangiectasia, Drug therapy, Pancreatic neoplasms, Prognosis

## Abstract

**Background:**

Loss of expression of the gene *ataxia-telangiectasia mutated* (*ATM*), occurring in patients with multiple primary malignancies, including pancreatic cancer, is associated with poor prognosis. In this study, we investigated the detailed molecular mechanism through which ATM expression affects the prognosis of patients with pancreatic cancer.

**Methods:**

The levels of expression of ATM and phosphorylated ATM in patients with pancreatic cancer who had undergone surgical resection were analyzed using immunohistochemistry staining. RNA sequencing was performed on *ATM*-knockdown pancreatic-cancer cells to elucidate the mechanism underlying the invlovement of ATM in pancreatic cancer.

**Results:**

Immunohistochemical analysis showed that 15.3% and 27.8% of clinical samples had low levels of ATM and phosphorylated ATM, respectively. Low expression of phosphorylated ATM substantially reduced overall and disease-free survival in patients with pancreatic cancer. In the pancreatic cancer cell lines with ATM low expression, resistance to gemcitabine was demonstrated. The RNA sequence demonstrated that *ATM* knockdown induced the expression of MET and NTN1. In *ATM* knockdown cells, it was also revealed that the protein expression levels of HIF-1α and antiapoptotic BCL-2/BAD were upregulated.

**Conclusions:**

These findings demonstrate that loss of ATM expression increases tumor development, suppresses apoptosis, and reduces gemcitabine sensitivity. Additionally, loss of phosphorylated ATM is associated with a poor prognosis in patients with pancreatic cancer. Thus, phosphorylated ATM could be a possible target for pancreatic cancer treatment as well as a molecular marker to track patient prognosis.

**Supplementary Information:**

The online version contains supplementary material available at 10.1186/s12885-023-11294-3.

## Background

Pancreatic cancer (PC) is a fatal disease of the digestive system characterized by high invasiveness and recurrence [1], and ranks seventh among the leading causes of global cancer-related deaths [2]. Owing to the absence of PC-related symptoms in early stages and rapid disease progression, micro-metastasis usually occurs by the time of clinical diagnosis. Less than 20% of patients can receive surgical treatment; even in cases of negative surgical margin, the tumor is prone to recurrence and metastasis post-surgery. Approximately 20–30% of patients only survive 1 year after surgical resection, with the 5-year survival rate being less than 9% [3–5]. Early metastasis and local invasion are critical factors for poor patient prognosis. The underlying mechanisms of PC are complex, including gene mutations that regulate the DNA damage response and repair (DDR) system, anti-apoptotic signal activation, epithelial-mesenchymal transition (EMT), and tumor-neuroglia that promote cancer metastasis through paracrine signaling interactions [6–8]. Currently, chemotherapy combined with surgical treatment improves patient prognosis. Gemcitabine has been used as a first-line drug for PC chemotherapy, but the survival benefit for patients remains very limited, mainly because most patients are gemcitabine-resistant, which affects its chemotherapeutic efficacy [4]. The abnormal activity of cell-cycle checkpoints, inactivation of apoptotic signals, and destruction of the gemcitabine metabolic pathway are associated with gemcitabine resistance [9]. Additionally, extensive research on PC has revealed that several germline mutations constitute high-risk factors for PC occurrence, some of which are efficient therapeutic targets for anticancer agents. Mutations in *Breast cancer gene 1* (*BRCA1*) and *BRCA2* are the most common causes of hereditary cancer syndrome, including PC, and are associated with high sensitivity to platinum and poly (ADP-ribose) polymerase (PARP) inhibitors [10]. Although great progress has been made in the treatment of PC, the complex molecular mechanisms involved in its prognosis and chemosensitivity remain to be fully elucidated.

Ataxia telangiectasia mutated (ATM), a key molecule in DDR system regulation, is associated with hereditary PC [6]. The *ATM* gene is located on chromosome 11q 22–23, contains 66 exons, and encodes a 350-kDa protein belonging to the phosphatidylinositol 3-kinase-related protein kinase family [11, 12]. ATM plays a vital role in repairing DNA double-strand breaks (DSBs), signaling at cell cycle checkpoints, and initiating apoptosis [11–14]. ATM loss intensifies the accumulation of reactive oxygen species (ROS), resulting in higher hydrogen peroxide levels [15, 16]. Under these circumstances, normal mitochondrial function is destroyed, a state which is closely related to tumor occurrence [16]. Germline mutations in *ATM* consitute the primary cause of autosomal recessive ataxia-telangiectasia (A-T) syndrome. Owing to this genomic instability, the risk of malignant tumors in patients with A-T is increased; approximately 25% of A-T patients develop cancer during their lifetime [17, 18]. In addition, *ATM* deletion can increase the accumulation of secondary mutations, thus forming a serious chain reaction and accelerating the process of A-T [18].

Undoubtedly, *ATM* mutations cause genomic instability and defects in DDR and cell-cycle checkpoint control, which promote resistance to apoptosis and occurrence and development of cancer. A large-scale study showed that approximately 5% of patients with tumors had a loss or mutation of *ATM* [19]. Compared with that of other mutations, ATM deficiency is more frequently observed in many tumors, including gastric cancer (21.4–63.9%), breast cancer (31%), and PC (11–24.5%) [20]. The main mechanism of ATM deficiency can be regarded as the target of epigenetic silencing, wherein *ATM* promoter hypermethylation causes a decrease in its protein levels [21]. Moreover, the loss of ATM is correlated with a low degree of tumor differentiation and notable increase in lymph-node metastasis, critically affecting patient survival [22]. Collectively, ATM deficiency may be associated with the progression of PC cells by disturbing cell-cycle checkpoint control and the apoptotic pathway, resulting in chemoresistance and poor prognosis in patients with PC.

## Materials and methods

### Patients and tissue samples

Tissue samples from 144 PC patients who underwent surgical resection from 2011 to 2018 at the Tohoku University Hospital were collected for immunohistochemistry (IHC) staining. Patient clinical data were collected from medical records. The institutional review board of Tohoku University (Ethics Committee Tohoku University Graduate School of Medicine, Sendai, Japan) authorized this study in 2021 (Approval No. 2021-1-864). The requirement for informed consent was waived, and an opt-out method was used because of the retrospective study design. The present study was conducted in accordance with the Declaration of Helsinki. TNM classification was performed according to the Union for International Cancer Control staging system.

### RNA isolation and quantitative real-time PCR (RT-qPCR) analysis

PC cell lines (SUIT-2 and MIA-PaCa2) were purchased from the American Type Culture Collection (ATCC, Manassas, VA, USA). Total RNA was isolated from PC cell lines using the Nucleospin RNA kit (Takara Biotechnology, Tokyo, Japan) according to the manufacturer’s protocol. Reverse transcriptase reactions were performed using the PrimeScript™ RT Master Mix (Cat. No. RR036A, Takara Biotechnology). TB Green Premix Ex Taq II and ROX plus (Cat. No. RR82LR, Takara Biotechnology) were used to amplify cDNA in a StepOne real-time PCR system (Thermo Fisher Scientific; Waltham, MA, USA). *GAPDH* was used as an internal control gene to normalize the transcriptional expression of target genes, and quantification was performed using the 2^−ΔΔCq^ method. Primer sequences used in this study are listed in Supplementary Table S1.

### Transfection with small interfering RNA (si-RNA)

siRNAs for human *ATM* (ATM-siRNA1: 5′-GCAAUU GUCAUAAAACCAAtt-3′; ATM-siRNA2: 5′-GCUGUUACCUG UUUGAAAAtt-3′; Thermo Fisher Scientific,) and Silencer Select Negative Control No. 1 siRNA (Cat. No. 4,390,843, Thermo Fisher Scientific) were transfected in cells using the Lipofectamine RNAiMAX Reagent (Cat. No. 13,778,150, Thermo Fisher Scientific) according to the manufacturer’s protocol.

### Western blotting

For western blotting, 18 µg of protein was loaded onto a 4–15% sodium dodecyl-sulfate polyacrylamide gel for electrophoretic separation (Cat. No. 4,561,085, Bio-Rad, Hercules, CA, USA). The primary antibodies used in this study are as follows: anti-ATM (1:2000; Cat. No. ab32420, Abcam, Cambridge, UK); anti-phospho S1981-ATM (1:50 000; Cat. No. ab81292, Abcam); anti-HIF1α (1:1000; Cat. No. ab51608, Abcam); anti-phospho-HIF1α (1:1000; Cat. No. AF0062, Funakoshi, Tokyo, Japan); anti-Met (1:1000; Cat. No. 3127, Cell Signaling Technology Danvers, MA, USA); anti-Netin1 (1:1000; Cat. No. ab126729, Abcam); Anti-Apoptosis Sampler Kit (anti-BCL-2: 1:1000; anti-BAD: 1:1000; Cat. No. E051004, Funakoshi). Anti-β-actin antibody (1:10 000; Cat. No. 66009-1, Proteintech, Tokyo, Japan) was used as a standardized internal reference to compare the target protein expression levels. Finally, immunoreactive bands were detected using the ChemiDoc Imaging System (Bio-Rad) and analyzed using ImageJ software (NIH, Bethesda MD, USA).

### Immunohistochemistry (IHC)

PC tissue samples were fixed in 10% formalin (FUJIFILM) and embedded in paraffin. Staining was performed on 4 μm-thick sections, which were incubated at 4 °C overnight with rabbit anti-ATM (1:100; Cat. No. ab32420, Abcam) or rabbit anti-phospho S1981-ATM (1:100; Cat. No. ab81292, Abcam) as the primary antibody. In non-neoplastic cells, ATM expression was observed in islets and lymphoid cells, whereas phospho-ATM expression was observed in nerve cells, both of which served as internal controls. As a negative control, normal rabbit IgG (Cat. No. IS600, Dako, CA, USA) was used instead of the primary antibodies. ATM and phospho-ATM expression in cancer cells was evaluated visually by two pathologists (KH and TF) and classified into three grades according to the intensity of staining: score 0, null expression; score 1, faint expression; score 2, evident expression. A faint expression (score 1) was considered when the staining intensity was lower than that in adjacent internal controls (normal islet cells or nerve cells) on the same section, whereas an evident expression (score 2) was considered when the staining intensity was higher than or equal to that in the internal controls. Low ATM/phospho-ATM expression was assigned a score of 0, whereas high ATM/phospho-ATM expression was assigned scores of 1 and 2.

### RNA sequencing (RNA-seq) and analysis

Total RNA extracted from PC cell lines was sent to the Beijing Genomics Institute for transcriptome library preparation and sequencing. After filtering using SOAPnuke [23], the clean reads were mapped to the reference genome (GCF_000001405.38_GRCh38.p12) using Hierarchical Indexing for Spliced Alignment of Transcripts (HISAT2) [24]. After genome mapping, Bowtie2 [25] was used to reconstruct transcripts, and RSEM [26] was used to calculate the gene expression level of each sample. Subsequently, the differentially expressed genes (DEGs) were identified using DEGseq [27]. DEGs were defined as genes with a fold change > 2, Qvalue < 0.05, and FDR < 0.001, which were screened as significant differentially expression genes, using a Venn diagram and Heat Map Analysis.

### Cell proliferation and cytotoxicity assays

The MTT assay was conducted to estimate cell proliferation. Briefly, after 72 h of transfection with siRNA, cells were seeded in 96-well plates and incubated for 0, 1, 2, 3, 4, and 5 days at 37 °C. For the gemcitabine cytotoxicity assay, the medium was replaced with an alternate medium containing different concentrations of gemcitabine and cultured for 120 h. After incubation, 20 µL of MTT solution (Promega, Madison, WI, USA) was added and incubated for 2 h. The number of surviving cells was estimated by measuring absorbance at an optical density (OD) of 490 nm. Each experiment was performed in triplicate.

### Wound-healing assay

The migration capability of PC cells was verified using a wound-healing assay. SUIT-2 and MIA-PaCa2 cells (6 × 10^5^ cells/well) were plated in 6-well plates and transfected with siRNA for 72 h. After reaching 100% confluence, the cells were scraped using sterile 10 µL pipette tips, and the PC cells were subsequently incubated in RPMI 1640 or DMEM supplemented with 10% FBS and 1% penicillin at 37 °C and 5% CO_2_. The wound area was recorded using a Zeiss optical microscope (Oberkochen, Germany), and changes in the wound area of each group were calculated using ImageJ software, using the following calculation formula: Wound-healing rate = (Area _time 0_ − Area _time measure_) /Area _time 0_. This assay was independently repeated thrice.

### Intracellular reactive oxygen species (ROS) assay

The amount of intracellular ROS was detected using an ROS activity assay kit (Fluorimetric Intracellular Total ROS Activity Assay Kit, AAT Bioquest, MA, USA) according to the manufacturer’s instructions, and fluorescence signals were measured using a Spectra MAX M2e microplate reader (Molecular Devices, CA, USA). MIA-PaCa2 and SUIT-2 were stimulated with 2 µg/mL lipopolysaccharide for 24 h as the positive control group.

### Statistical analysis

Statistical analyses were performed using JMP software version 14 (SAS Institute Inc., Cary, NC, USA). Survival curves were estimated using the Kaplan–Meier method to compare overall survival (OS) and disease-free survival (DFS), and statistical significance was determined using the log-rank test. Data are expressed as the mean ± standard deviation (SD). To evaluate the factors associated with prognosis in multivariate analysis, a Cox proportional hazards regression model was used. Statistical significance was set at *p* < 0.05.

## Results

### Patient characteristics and ATM or phospho-ATM expression on prognosis

**Fig. 1:**
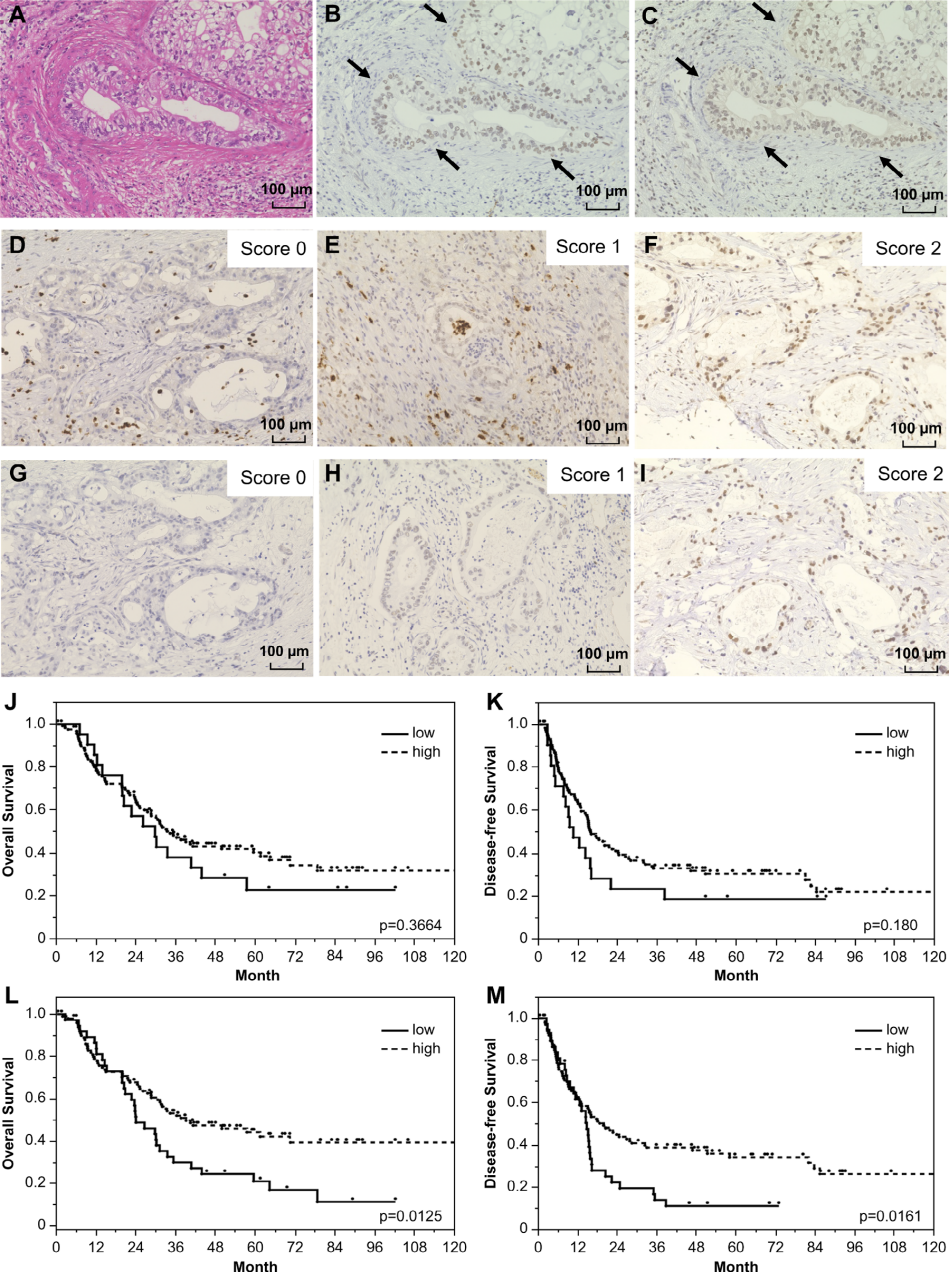
ATM/p-ATM expression and impact on prognosis of PC patients. **A**, PC stained with hematoxylin and eosin. **B**, ATM immunoreactivity was detected in PC cell nucleus (arrow), (same area as Fig. 1A). **C**, P-ATM immunoreactivity was detected in PC cell nucleus (arrow), (same area as Fig. 1A). Representative images of ATM expression evaluated using a score of 0 (**D**), 1 (**E**), or 2 (**F**). Phospho-ATM expression is scored as 0 (**G**), 1 (**H**), or 2 (**I**). Log-rank test survival analyses of correlation between ATM expression and OS (**J**) and DFS (**K**) in PC patients. Log-rank test survival analysis of correlation between p-ATM expression and OS (**L**) and DFS (**M**) in PC patients. IHC, immunohistochemistry; PC, pancreatic cancer; ATM, ataxia telangiectasia mutated; p-ATM, phosphorylated ATM; OS, overall survival; DFS, disease-free survival

A total of 144 patients with stage IA-IIB PC were analyzed for ATM and phospho-ATM expression using immunohistochemistry. The basic clinicopathological features of all participants are detailed in Supplementary Table S2. Their age range was 39–88 years (median age: 70 years), and 82 (56.9%) cases were male, whereas 62 (43.1%) cases were female. Both ATM and phospho-ATM immunoreactivity was detected in the nucleus of the patients’ PC cells (Fig. 1A-C). The expression pattern of these molecules showed diffused staining in the PC tissues (Fig. 1D-I). The expression levels were much lower in the surrounding, normal pancreatic tissue than in cancer cells. Among the 144 patients with PC, high and low ATM expression levels were observed in 122 (84.7%) and 22 (15.3%) cases, respectively. Moreover, phospho-ATM high and low expression levels were observed in 104 (72.2%) and 40 (27.8%) cases, respectively. The incidence of low phospho-ATM expression was relatively higher (15.3% vs. 27.8%). In this study, ATM and phospho-ATM expression had no significant effect on tumor size, stage, and CA 19 − 9 before and after surgery. Regarding the differentiation of cancer cells, patients with low ATM expression had a higher tendency for the poorly differentiated type, but no statistical difference was observed. The Kaplan–Meier analysis of patient survival revealed that ATM expression had no effect on OS (*p* = 0.3664) and DFS (*p* = 0.1800; Fig. 1J, K). However, the level of phospho-ATM significantly affected OS and DF; when compared to patients with high phospho-ATM levels, those with low phospho-ATM levels exhibited significantly worse OS (*p* = 0.0125) and DFS (*p* = 0.0161; Fig. 1L, M).

### Low ATM expression stimulated PC cell proliferation and migration

**Fig. 2:**
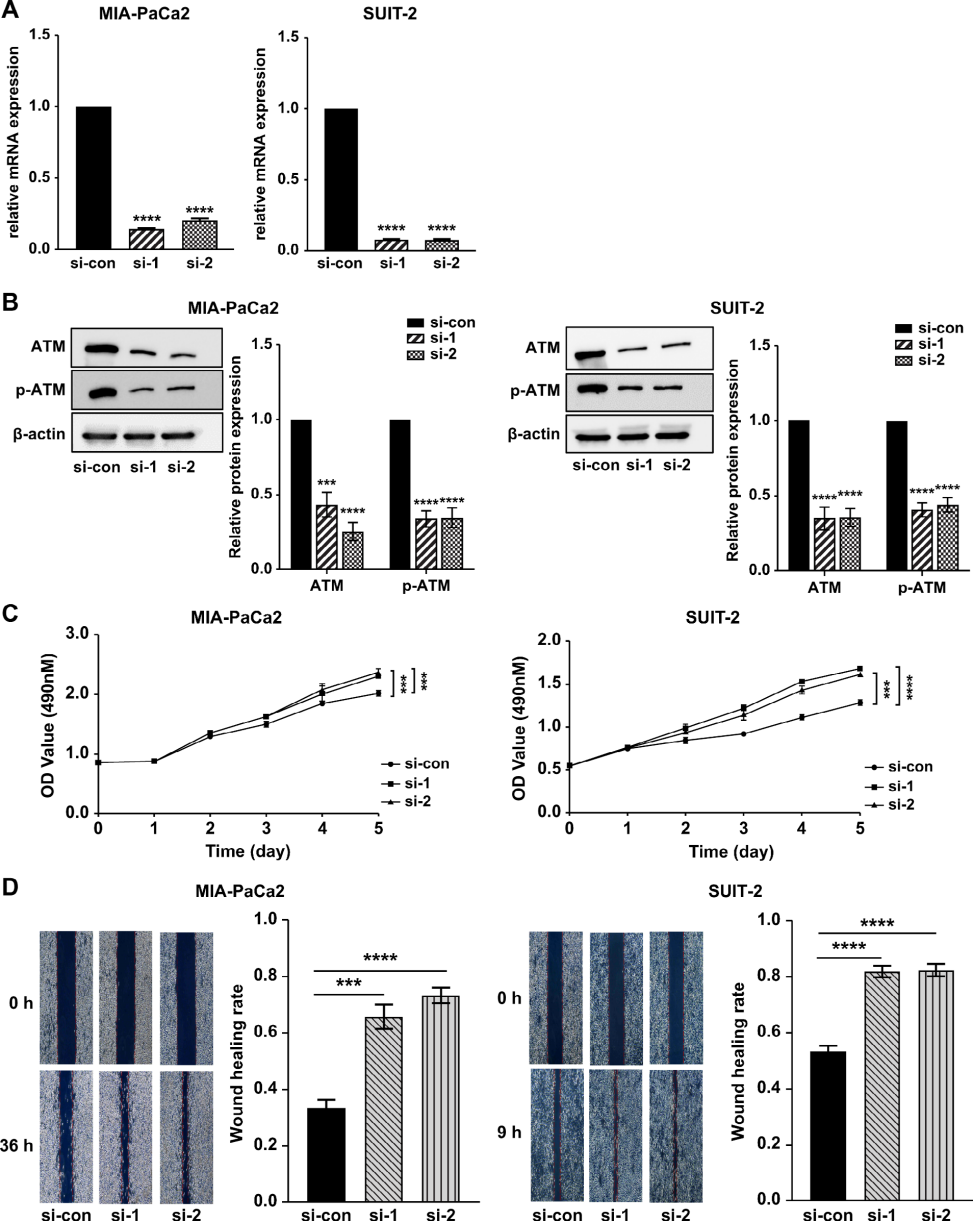
Expression of ATM in PC cell lines. RT-qPCR analysis of *ATM* mRNA expression after transfection with si-ATM and si-ATM negative control, respectively, in PC MIA-PaCa2 (**A**; left) and SUIT-2 (**A**; right) cell lines. After transfection with si-ATM and si-ATM negative control, ATM and p-ATM expression in MIA-PaCa2 (**B**; left) and SUIT-2 (**B**; right) cells was measured by western blotting and quantified using ImageJ software. For the original bands, please see additional file Supplementary Fig. S2. Cell-proliferation assay using MIA-PaCa2 (**C**; left) and SUIT-2 cells (**c**; right). Migration activities were assessed using a wound-healing assay (**D**). All data are presented as mean ± SD from three independent experiments. ****p < 0.0001. PC, pancreatic cancer; ATM, ataxia telangiectasia mutated; p-ATM, phosphorylated ATM. si-con/si-ATM1/si-ATM2, si-ATM RNA negative control/1/2

To identify the role of ATM in the regulation of proliferation and migration, MIA-PaCa2 and SUIT-2 cells were transfected with an ATM si-RNA or negative control for 72 h, and the successful knockdown of *ATM* was confirmed (Fig. 2A, B). In addition, it was demonstrated that the expression of phospho-ATM was suppressed in ATM si-RNA transfected cells, and the reduction ratio in phospho-ATM expression was roughly equivalent to that of ATM expression (Fig. 2A, B). MTT assays were performed to detect the effect of low ATM expression on PC cell proliferation. Compared with the negative-control group, low ATM expression stimulated tumor-cell proliferation (Fig. 2C). Further, wound-healing assays were performed to determine whether ATM affects cell migration. Wound areas of MIA-PaCa2 cells transfected with either ATM si-RNA1 (*p* < 0.001) or ATM si-RNA2 (*p* < 0.0001) were significantly lower than those of MIA-PaCa2 control cells (Fig. 2D). Similar results were obtained in SUIT-2 cells transfected with either ATM si-RNA1 (*p* < 0.0001) or ATM si-RNA2 (*p* < 0.0001; Fig. 2D). Therefore, low ATM expression promotes PC cell line proliferation and migration.

### Identification of DEGs in ATM-silenced PC cell lines

**Fig. 3:**
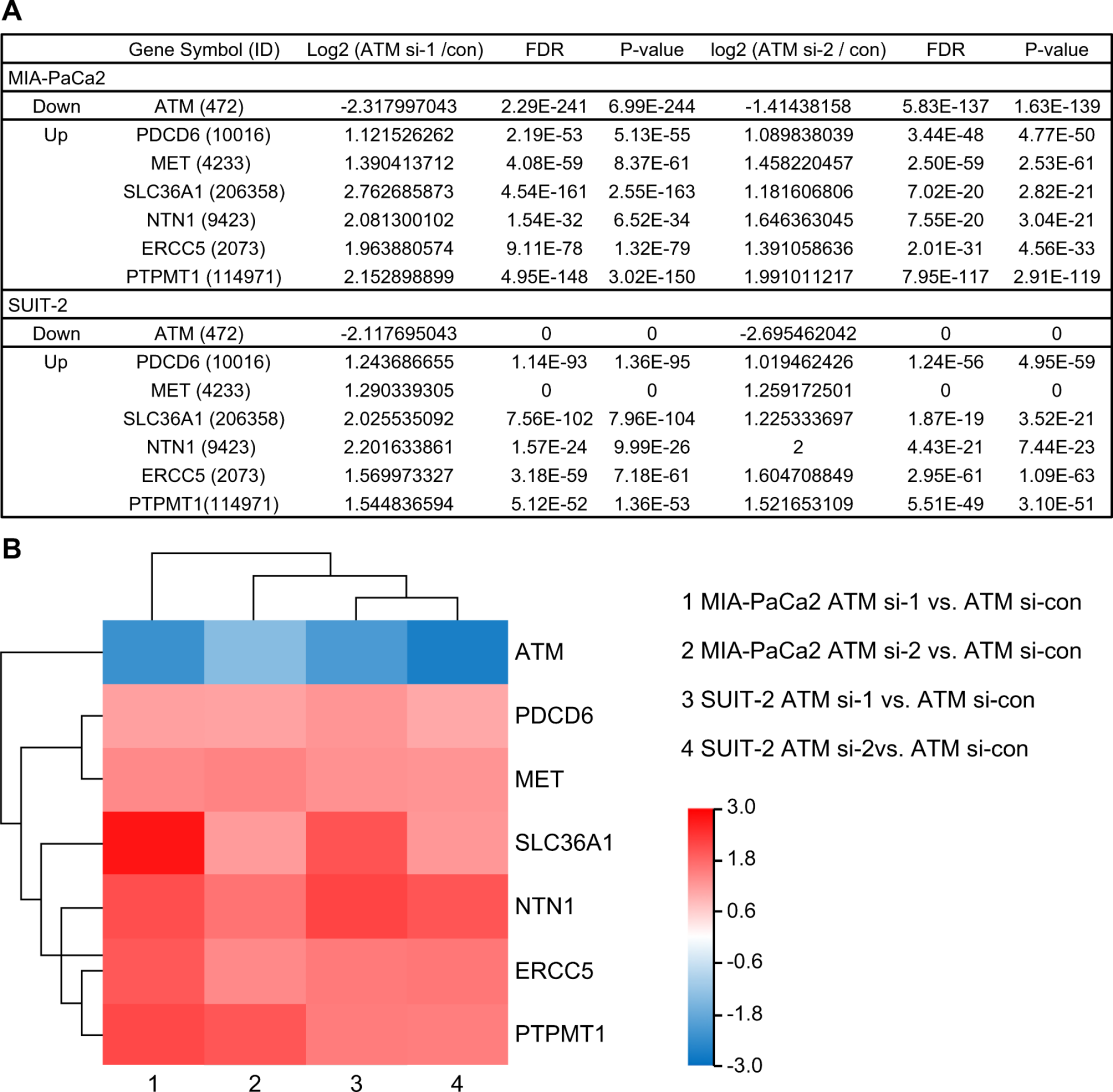
RNA sequencing in *ATM*-knockdown PC cell lines. RNA sequencing was performed after transfection of PC cell lines with si-ATM and si-ATM-negative controls. (**A**) Common DEGs within a unique cluster of the four groups from the two cell lines were listed. DEGs were defined as genes with fold change > 2, p-value < 0.05, and FDR < 0.001. (**B**) Heatmap analysis of seven DEGs in two cell lines. PC, pancreatic cancer; ATM, ataxia telangiectasia mutated; DEGs, differentially expressed genes

To explore the detailed mechanism of *ATM* knockdown on downstream genes, RNA sequencing was performed on *ATM*-knockdown MIA-PaCa2 and SUIT-2 cells. DEGs were identified by comparing the gene expression between four groups (“ATM si1 vs. control si” and “ATM si2 vs. control si” in MIA-PaCa2 and in SUIT-2). Among the identified DEGs in the four groups, seven genes (*ATM, PDCD6*, *MET*, *SLC36A1*, *NTN1*, *ERCC5*, and *PTPMT1*) that were common in all four groups are listed in Fig. 3A. The Heat Map Analysis demonstrated that there were no down-regulated genes other than *ATM*, and the other six genes were strongly up-regulated (Fig. 3B). Kyoto Encyclopedia of Genes and Genomes (KEGG) pathway enrichment analysis was performed on the screened DEGs, and cancer-related pathways, including the apoptotic pathway, was identified as significant (*p* < 0.05; Supplementary Figure S1) [28].

### ATM silencing up-regulated the expression MET, NTN1, and antiapoptotic protein in PC cell lines

**Fig. 4:**
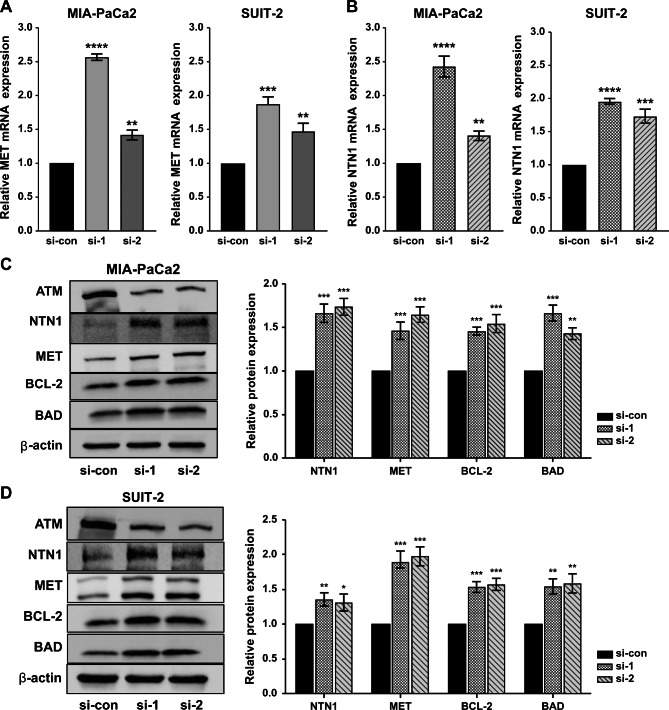
HIF-1α, NTN1, BCL-2, and BAD expression in *ATM*-knockdown PC cell lines. RT-qPCR analysis of *MET* mRNA expression after transfection with si-ATM and si-ATM negative control in MIA-PaCa2 (**A** left), and SUIT-2 (**A** right) cells. RT-qPCR analysis of *NTN1* mRNA expression after transfection with si-ATM and si-ATM negative control in PC cell lines, MIA-PaCa2 (**B** left), and SUIT-2 (**B** right) cells. NTN1, MET, BCL-2, and BAD expression after *ATM*-knockdown was measured by western blotting and quantified using ImageJ software in MIA-PaCa2 (**C**), and SUIT-2 (**D**) cells. For the original bands, please see additional files Supplementary Fig. S3 and Fig. S4. All data are presented as mean ± SD from three independent experiments. *p < 0.05; **p < 0.01; ***p < 0.001; ****p < 0.0001. PC, pancreatic cancer; ATM, ataxia telangiectasia mutated; si-con/si-1/si-2, ATM si-RNA negative control/1/2; MET, mesenchymal-epithelial transition; NTN1, netrin-1; BCL-2, B cell lymphoma-2; BAD, Bcl-2 agonist of cell death

In recent years, several studies have demonstrated that the up-regulation of MET and NTN1 was closely related to the occurrence and development of various malignancies including PC [29, 30]. To confirm the effect of *ATM* knockdown on *MET* and *NTN1* mRNA expression, RT-PCR was performed. *MET* and *NTN1* mRNA expression was substantially up-regulated in *ATM*-knockdown PC cell lines (Fig. 4A, B). In addition, increased MET and NTN1 expression was confirmed using western blotting (Fig. 4C, D). MET can inhibit the apoptotic pathway by inducing anti-apoptotic BCL-2 family [29]. Therefore, *ATM* silencing markedly strengthened BCL-2 and BAD protein levels in MIA-PaCa2 and SUIT-2 cells (Fig. 4C and D).

### ATM silencing induces ROS accumulation and hypoxia inducible factor-1α (HIF-1α) expression in PC cell lines

**Fig. 5:**
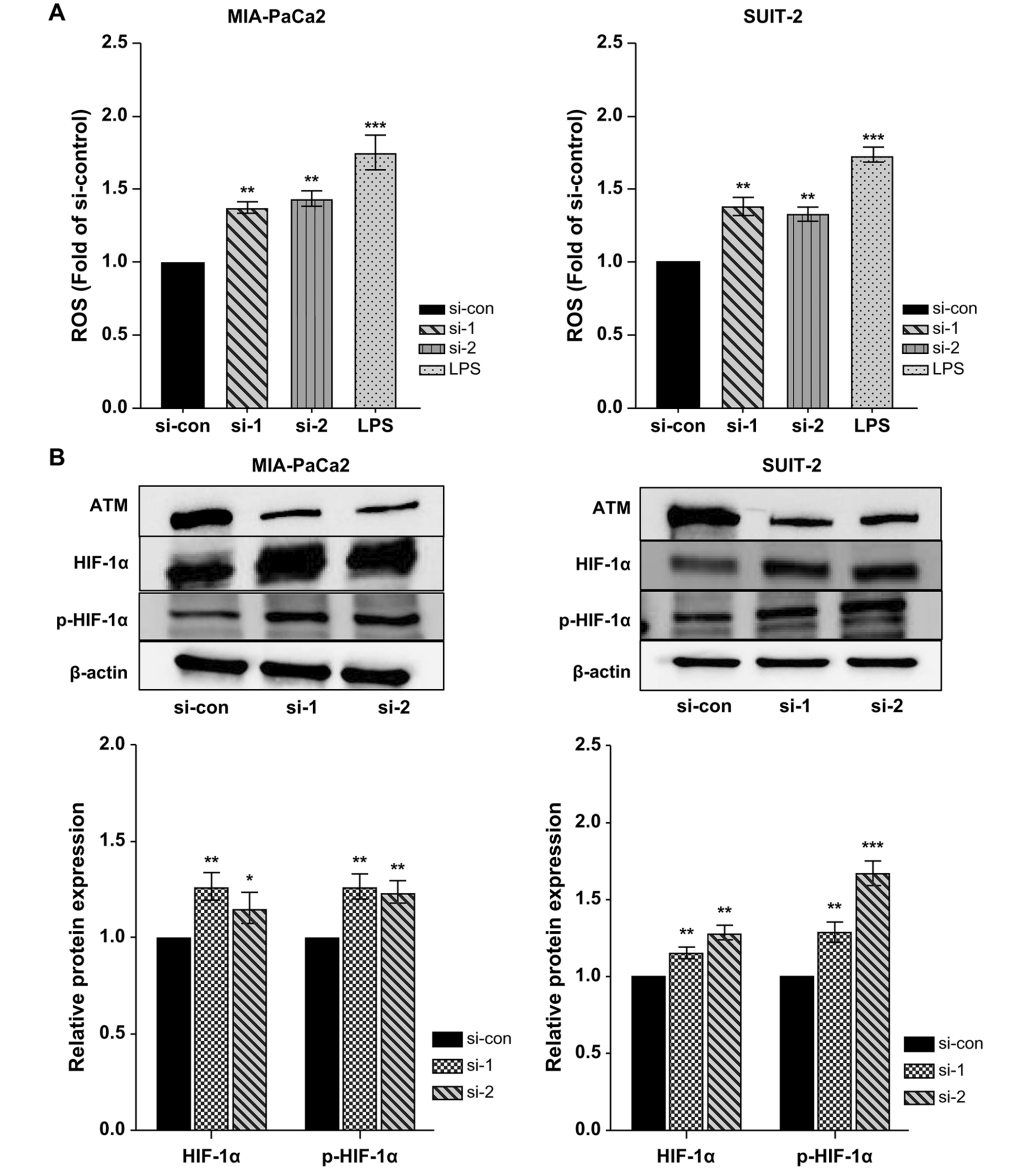
ATM silencing induces ROC accumulation and HIF-1α expression in PC cell lines. Intracellular ROS levels were measured after transfection with si-ATM and si-ATM negative control in PC cell lines, MIA-PaCa2 (**A**; left), and SUIT-2 (**A**; right) cells. Cells treated with 2 µg/mL lipopolysaccharide for 24 h were used as positive control. HIF-1α and p-HIF-1α expression after *ATM*-knockdown in MIA-PaCa2 (**B**; left) and SUIT-2 (**B**; right) was measured by western blotting and quantified using ImageJ software. For the original bands, please see additional files Supplementary Fig. S5 and Fig. S6. All data are presented as mean ± SD from three independent experiments. *p < 0.05; **p < 0.01; ***p < 0.001; ****p < 0.0001. PC, pancreatic cancer; ATM, ataxia telangiectasia mutated; si-con/si-1/si-2, ATM si-RNA negative control/1/2; HIF-1α, hypoxia inducible factor-1α; p-HIF-1α, phosphorylated hypoxia inducible factor-1α

ATM deficiency can induce ROS accumulation and stable HIF-1α expression by disturbing mitochondrial functions. To confirm the effect of *ATM* knockdown on intercellular ROS accumulation in PC cell lines, the ROS activity assay was performed. *ATM* knockdown increased intercellular ROS activities in MIA-PaCa2 and SUIT-2 cells (Fig. 5A). In addition, HIF-1α and phospho-HIF-1α expression was up-regulated in *ATM*-knockdown MIA-PaCa2 and SUIT-2 cells (Fig. 5B).

### ATM expression can regulate PC cell sensitivity to gemcitabine

**Fig. 6:**
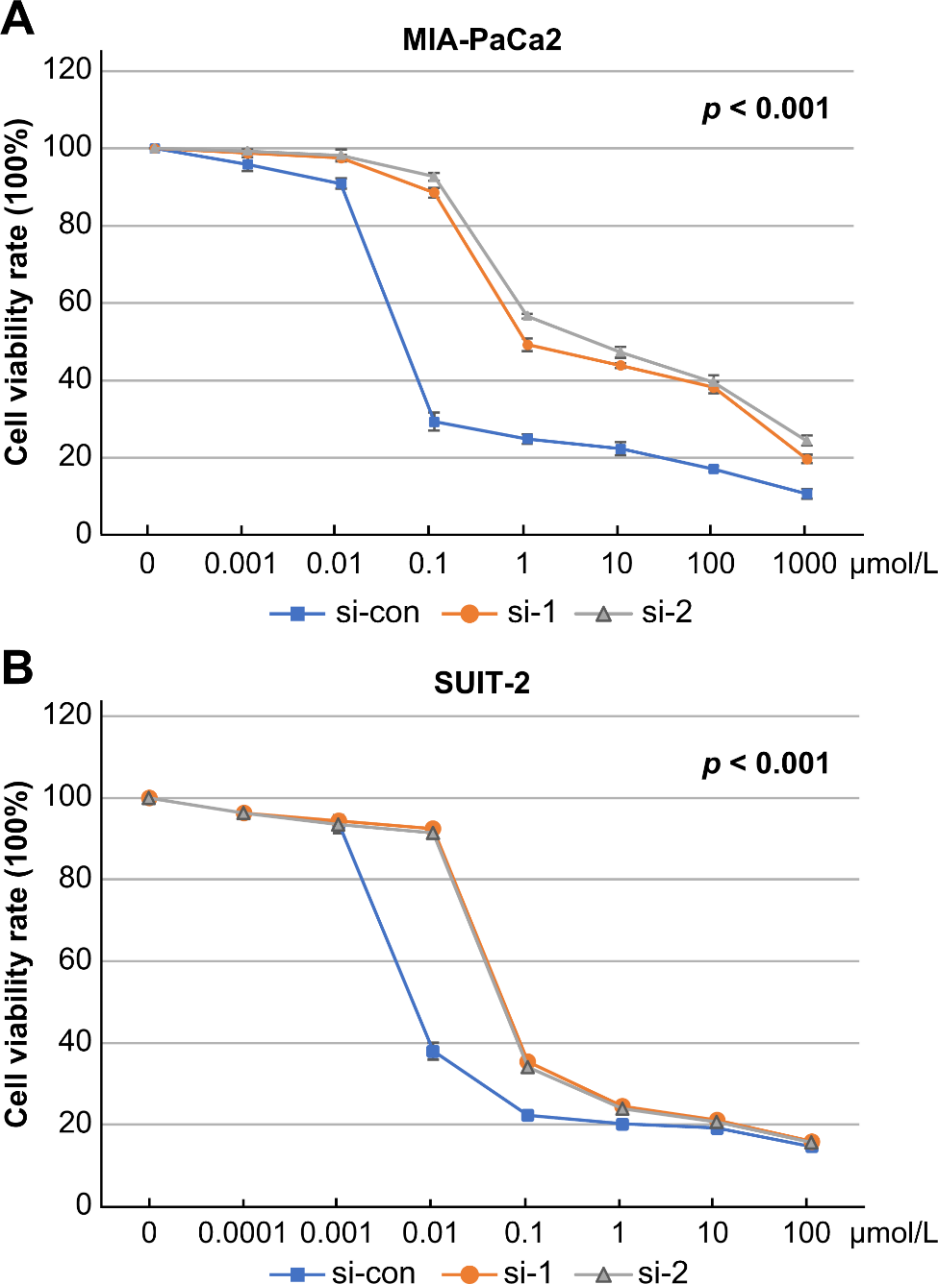
Gemcitabine chemosensitivity in *ATM*-knockdown PC cell lines. Cytotoxicity was assessed using the MTT assay in MIA-PaCa2 and SUIT-2 cells. Differences in IC_50_ between ATM si-RNA (1&2) and ATM si-RNA con were compared; effects of ATM expression levels on gemcitabine sensitivity were calculated. MIA-PaCa2, IC_50_: con vs. si-1, *p* < 0.001; con vs. si-2, *p* < 0.001. SUIT-2, IC_50_: con vs. si-1, *p* < 0.001; con vs. si-2, *p* < 0.001. PC, pancreatic cancer; ATM, ataxia telangiectasia mutated; si-con/si-ATM1/si-ATM2, si-ATM RNA negative control/1/2

We evaluated whether ATM expression levels affect gemcitabine cytotoxicity in PC cell lines. Post-transfection with an ATM siRNA, the IC_50_ of MIA-PaCa2 and SUIT-2 cells significantly increased when compared with that of the control group (*p* < 0.001 and *p* < 0.001, respectively; Fig. 6). Therefore, *ATM* knockdown suppresses gemcitabine sensitivity in PC cell lines.

## Discussion

ATM plays a unique role in DDR, cell-cycle checkpoint activation, maintenance of genomic stability, and apoptotic system regulation [18]. Loss of ATM results in a greater risk of developing tumors; therefore, this can be used as an important observational risk factor in cancer progression. Previously, it was reported that the loss of ATM and normal TP53 expression is associated with poor prognosis in PC patients [31]. Likewise, ATM expression can be used as a prognostic marker in breast, gastric, and gall bladder cancers [20, 32, 33]. Although we did not obtain any evidence that ATM loss has an impact on PC patient prognosis, we are the first to report that low phospho-ATM expression is significantly important for OS and DFS in PC patients. Phospho-ATM loss is associated with early-stage tumor progression, tumor growth, and melanoma prognosis [34]. In addition, genomic studies have confirmed that ATM plays an important role as a part of intracellular signaling networks when autophosphorylated at serine 1981 (Ser1981) and transformed from an inactive homodimer to an active monomer; interestingly, ATM activity is regulated by phosphorylated ATM [7, 19, 21].

Our gene-expression analysis revealed the upregulation of *MET* and *NTN1* after *ATM* knockdown. Therefore, we hypothesized the occurrence of a disorder in redox reactions in ATM-deficient cells, which in turn would regulate HIF-1α activity and participate in *MET* and *NTN1* transcriptional regulation via HIF-1α. ATM is expressed in the nucleus and cytoplasm and is present in the mitochondria [35], where it plays a positive role in regulating metabolism and maintaining homeostasis of the internal environment. Mitochondria are the main sites of ROS synthesis. ATM promotes activation of G6PDH, the rate-limiting enzyme of the pentose phosphate pathway (PPP), thereby regulating PPP [19, 36–38]. The main function of ATM is to participate in oxidative-stress regulation and maintain oxidative stress in a reduced state to avoid damage caused by excessive ROS accumulation. ATM deficiency has been reported to result in increased ROS accumulation [35, 39]. In addition, mitochondrial ROS plays a pivotal role in HIF-1α activation and regulates HIF-1α expression through several signaling pathways [40–42]. Some studies have confirmed that oxidative stress is induced in ATM-deficient cells. More importantly, the absence of ATM stimulates HIF-1α expression through post-translational modifications [43]. Here, we demonstrated that *ATM* knockdown in cells via siRNA transfection led to intercellular ROS accumulation and consistently high HIF-1α and phospho-HIF-1α expression. ATM loss in PC cells may increase oxidative stress and ROS accumulation, thereby causing increased HIF-1α and phospho-HIF-1α expression through post-transcriptional modifications.

As a transcriptional factor, HIF-1α binds to hypoxia-response elements (HREs) in the promoters of many genes, activating the mRNA transcription of various targets. HIF-1α binds to multiple sites in the promoter region of *MET*, regulating its transcription and increasing *MET* mRNA levels [44, 45]. Consecutively, MET kinase activation produces an amplification effect through tyrosine autophosphorylation and intermolecular interactions [46]. Continuous activation and amplification of the MET signal through the positive feedback pathway results in excessive *MET* transcription and translation [45]. Additionally, HIF-1α plays an indispensable role in inducing *NTN1* transcription [47, 48]. HIF-1α regulates *NTN1* transcription by binding to HREs in the promoter, amplifying its mRNA by more than 4-fold [47, 48]. Here, RT-qPCR analyses indicated that ATM loss up-regulates *MET* and *NTN1* transcription. The activation of HIF-1α by *ATM* silencing may stimulate *MET* and *NTN1* transcriptional activity. Accordingly, MET and NTN1 protein levels significantly increased in *ATM*-knockdown cells.

MET plays a crucial role in tumor-cell proliferation, motility, anti-apoptosis, and angiogenic-factor secretion [49]. Moreover, its stem-like function is necessary for tumor initiation, propagation, and dissemination [50]. High MET expression levels have been observed in various carcinomas. In colorectal cancer, stromal myofibroblasts were shown to promote MET expression by secreting hepatocyte growth factor and activating WNT self-renewal to maintain long-term tumor stem-cell proliferation [50]. Similar conditions were observed in PC patients; tumor cell-derived fibroblasts promoted high MET expression, thereby enhancing their invasive ability through the basement membrane [49]. *NTN1*, as an oncogene, performs a homogeneous role in tumorigenesis similar to that of *MET*. NTN1 overexpression in mouse colons inhibits intestinal epithelial cell apoptosis, inducing colonic hyperplasia and adenoma, and demonstrates an upward trend. However, NTN1 may play an important role in the early stages of adenoma progression into colon cancer [51]. In addition, MET signaling activation has been related to phosphorylation of intracellular downstream signaling cascades, which are directly mediated by PI3K/AKT/NF-κB, MAPK/ERK, and FAK pathways or other possible indirect pathways, affecting the anti-apoptotic system, especially the up-regulation of the anti-apoptotic gene, *BCL-2* [29, 52]. Interestingly, NTN1 was found to stimulate BCL-2 expression. In this study, we confirmed MET and NTN1 signal activation due to the lack of ATM and observed significant differences in *ATM*-knockdown cell proliferation and migration abilities when compared with the control group. We further found that the anti-apoptotic signal was activated, and BCL-2 family (BCL-2, BAD) expression levels were enhanced, as indicated by western blot analysis. BAD (Bcl-2-associated death promoter), a pro-apoptotic protein belonging to the BCL-2 family, usually forms a dimer complex with BCL-2, triggering the activation of apoptotic signaling. Furthermore, BAD resists the G0/G1 phase and then enters the S phase, which explains its role in the acceleration of the cell cycle and promotion of proliferation [53]. In addition, BAD promotes mitochondrial metabolism by increased ROS generation, which in turn promotes the accumulation of BAD [54]. However, when phosphorylated BAD is separated from the complex, and apoptosis is suppressed [55]. Studies have also found that BAD expression promotes tumor-cell growth [53, 54]. Our findings confirmed that *ATM* knockdown in PC cell lines induces BAD expression. Overall, ATM deficiency increased PC cell proliferation through BAD signaling. Anti-apoptotic gene overexpression significantly contributes to tumor drug resistance due to their insensitivity to chemotherapeutic drugs. BCL-2 has been reported to inhibit tumor cell apoptosis induced by chemotherapy drugs by reducing the permeability of the mitochondrial membrane and maintaining its integrity [56]. Cytotoxicity assays verified that *ATM* knockdown reduced PC cell sensitivity to gemcitabine by up-regulating BCL-2 expression.

The present study has several limitations. First, RNA sequencing confirmed that four genes other than *MET* and *NTN1* were upregulated after *ATM* knockdown; however, we only focused on *MET* and *NTN1*. MET and NTN1 expression in PC cells is associated with high malignancy and poor prognosis [29, 30]. Thus, further studies are needed to explore the importance of the other four upregulated genes in *ATM*-knockdown PC cells. Second, although immunohistochemical and clinical data analyses showed that low phospho-ATM expression was associated with patient prognosis, the expression of phospho-ATM could not be suppressed directly in gemcitabine cytotoxicity assays. However, it was demonstrated that ATM-specific siRNA could suppress the phospho-ATM level to a level almost equivalent to that of ATM. Therefore, it was suggested that the reduced expression of ATM and its phosphates is significantly involved in cancer malignancy and gemcitabine resistance. To elucidate the molecular mechanisms, MET and NTN1, the expression of which is upregulated in *ATM* knockdown cells, needs to be investigated further. In addition, in vivo experiments using mouse xenograft models should be conducted.

In summary, clinical data and immunohistochemical analyses revealed that low ATM and phospho-ATM expression is frequently observed in PC patients, contradicting the results obtained in previous studies with larger patient cohorts. Interestingly, in our study, ATM deficiency had no effect on patient prognosis. However, our study demonstrated a strong relationship between phospho-ATM expression, PC progression, and patient survival, i.e., patients with low phospho-ATM expression have a poor prognosis. Furthermore, the present study is the first to clearly analyze the complex molecular mechanism of poor prognosis in patients with low ATM expression. Collectively, our study demonstrated that low ATM expression can up-regulate downstream oncogene expression, inhibit apoptosis, and promote PC cell proliferation and migration, especially of gemcitabine-resistant cells. Therefore, phospho-ATM expression is significant for PC patient prognosis, and ATM can be used as a potential therapeutic target for PC.

### Electronic supplementary material

Below is the link to the electronic supplementary material.

Supplementary Material 1

## Data Availability

Generated RNA-sequencing datasets were deposited in the Gene Expression Omnibus database (Accession No. GSE224882, https://www.ncbi.nlm.nih.gov/geo/query/acc.cgi?acc=GSE224882) All other supporting data are available from the corresponding authors on reasonable request.

## List of abbreviations

A-T: ataxia telangiectasia
ATM: Ataxia telangiectasia mutated
BAD: Bcl-2 agonist of cell death
BCL-2: B cell lymphoma-2
BRCA: Breast cancer gene
CHK2: Checkpoint kinase 2
DDR: DNA damage response and repair
DEGs: differentially expressed genes
DFS: disease-free survival
DSBs: DNA double strand breaks
DSBs: DNA double strand breaks
EMT: epithelial-mesenchymal transition
HIF-1α: hypoxia inducible factor-1α
HREs: hypoxia-response elements
IHC: immunohistochemistry
MET: mesenchymal-epithelial transition
NTN1: netrin-1
OD: optical density
OS: overall survival
PARP: poly (ADP-ribose) polymerase
p-ATM: phosphorylated ATM
PC: pancreatic cancer
PIKK: phosphatidylinositol 3-kinase-related protein kinase
PPP: pentose phosphate pathway
ROS: reactive oxygen species
SD: standard deviation
si-RNA: small interfering RNA
