# Supplementary material for: Reduced expression of phosphorylated ataxia-telangiectasia mutated gene is related to poor prognosis and gemcitabine chemoresistance in pancreatic cancer
Source: BMC Cancer. 2023 Sep 6;23:835. doi: 10.1186/s12885-023-11294-3 (PMC10481509; doi:10.1186/s12885-023-11294-3)
Supplement: Supplementary file 1 — Supplementary Material 1 [file 12885_2023_11294_MOESM1_ESM.pdf]

Supplementary Information

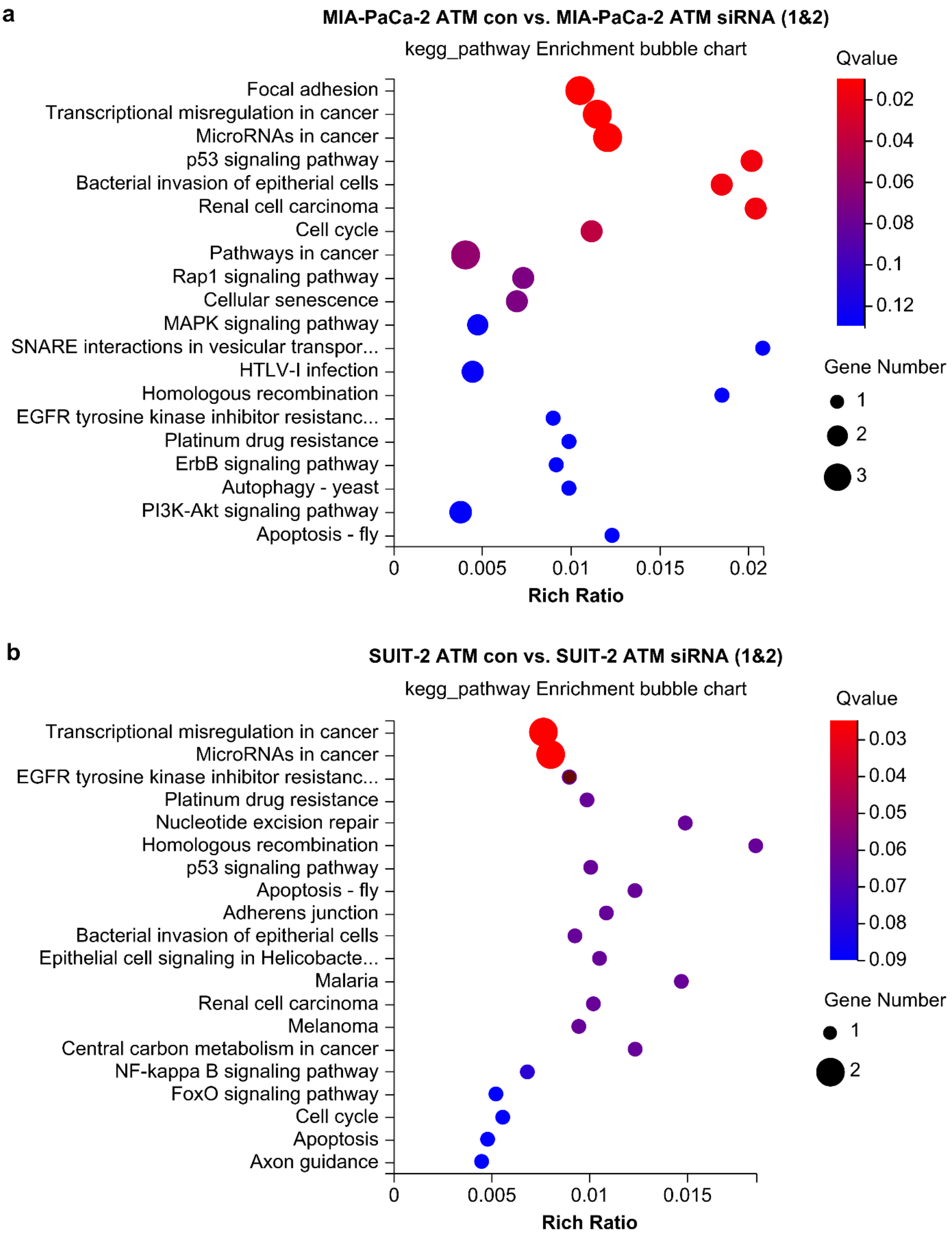

Supplementary Figure S1. Enrichment bubble diagram in KEGG pathway analysis.

Enrichment of KEGG pathways among different groups (A and B). Number of genes annotated to KEGG pathways is represented by bubble size. X- and y-axes represent the enrichment ratio and KEGG pathways, respectively. Color represents enriched significance. ATM, ataxia telangiectasia mutated; con/si-ATM1/si-ATM2, si-ATM RNA negative control/1/2.

Supplementary Table S1. Primer sequences used herein

Primer	Forward: 5′→3′	Reverse: 5′→3′
ATM	ATAGATTGTGTAGGTTCCGATGG	CATCTTGTC TCAGGTCATCACG
MET	AGCGTCAACAGAGGGACCT	GCAGTGAACCTCCGACTGTATG
NTN1	ACAACCCGCACAACCTGAC	GGGACAGTGTGAGCGTGAC
GAPDH	GCACCGTCAAGGCTGAGAAC	TGGTGAAG ACGCCAGTGGA

Supplementary Table S2. Clinicopathological characteristics of ATM and p-ATM expression in patients

		ATM			p-ATM		
		high<br>n=122	Low<br>n=22	p	High<br>n=104	Low<br>n=40	p
Age(years)	Median (range)	70(39–88)	68(42–85)	0.33	76(39–85)	72(48–88)	0.343
Gender	Male: Female	68:54	13:9	0.77	57:47	24:16	0.574
Pre-treatment CA19-9(U/ml)	Median (range)	76.5(1.6–3716)	184.6(10.1–10239)	0.025	88.5(6.7–3146)	81.4(1.6–10239)	0.655
Post-treatment CA19-9(U/ml)	Median (range)	19.6(0.6–1134)	16.7(3.9–426.9)	0.401	19.9(0.8–1134)	18.4(0.6–426.9)	0.641
Tumor size (mm)	Median (range)	25(4–70)	30(15–45)	0.252	25(4–70)	28(10–53)	0.059
UICC-T	1,2	36(29.5%)	4(18.2%)	0.887	30(28.8%)	10(25%)	0.97
	3	86(70.5%)	18(81.8%)		74(71.2%)	30(75%)	
UICC-N	0	44(36.1%)	8(36.4%)	0.796	37(35.6%)	15(37.5%)	0.587
	1	78(63.9%)	14(63.6)		67(64.4%)	25(62.5%)	

Postoperative Gemcitabine	yes	53(43.4%)	16(72.7%)	0.011	47(45.2%)	22(55.0%)	0.180
Tumor differentiation	tub1	24(19.7%)	1(4.5%)	0.127	19(18.3%)	5(12.5%)	0.275
	tub2	92(75.4%)	19(86.5%)		79(75.9%)	33(82.5%)	
	por	6(4.9%)	2(9.0%)		6(5.8%)	2(5%)	

Full-length gels and blots

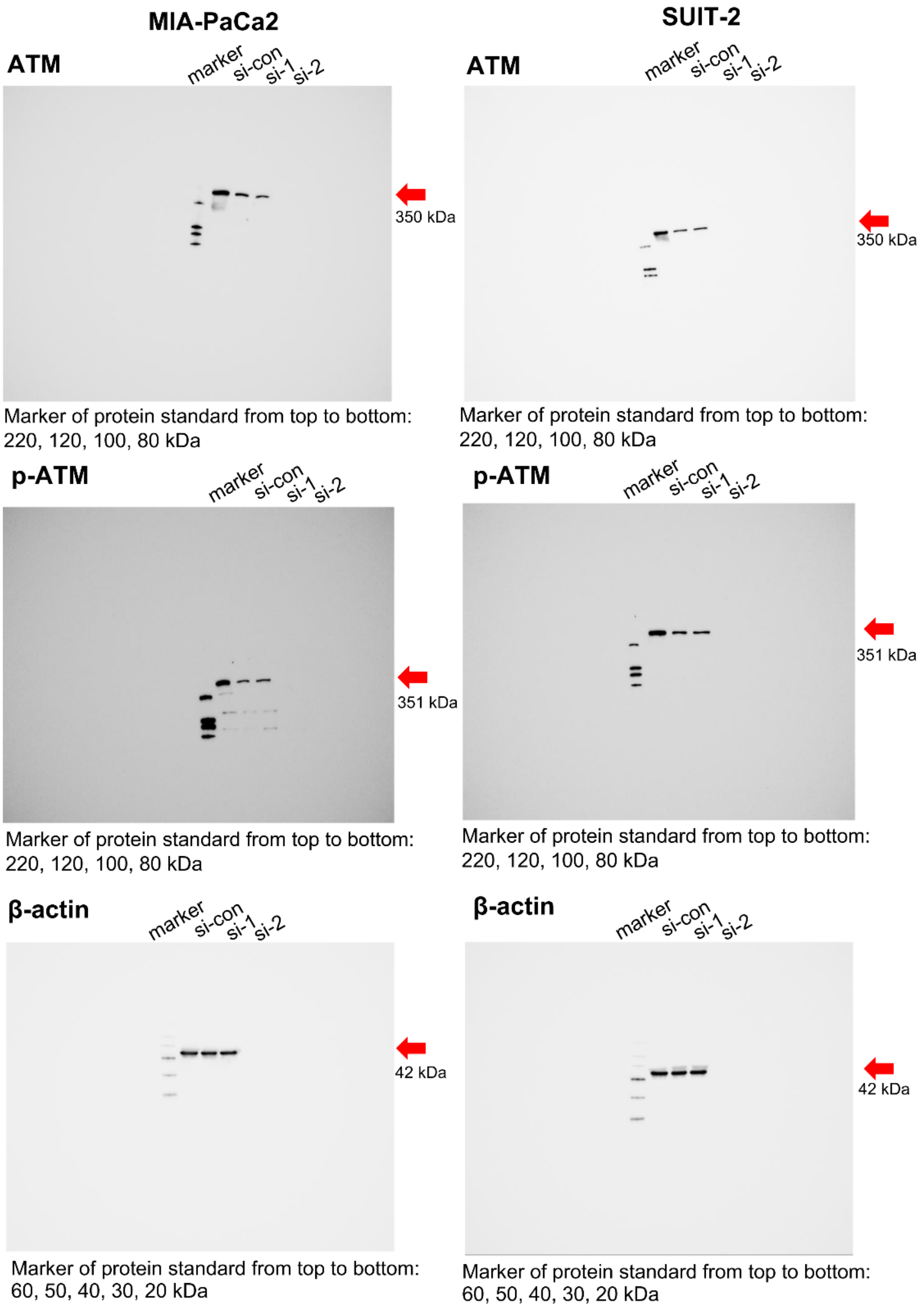

Supplementary Figure S2. Uncropped gel image for Figure 2

ATM

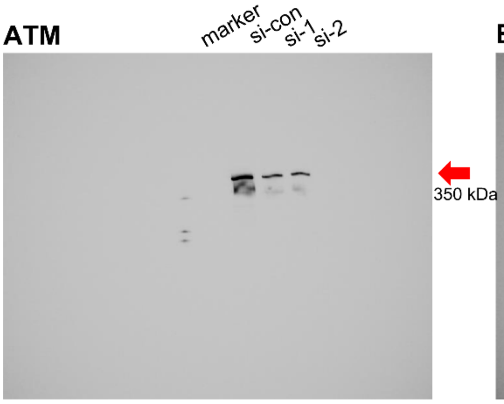

Marker of protein standard from top to bottom:
220, 120, 100, 80 kDa

BCL-2

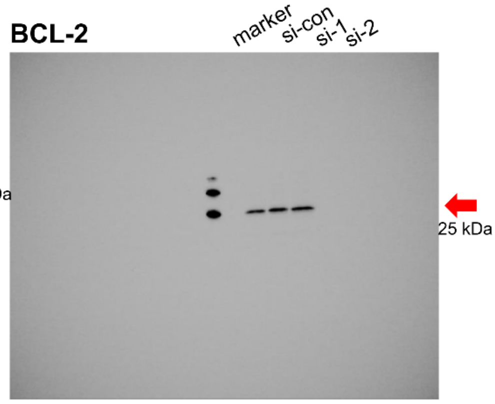

Marker of protein standard from top to bottom:
30, 20 kDa

NTN-1

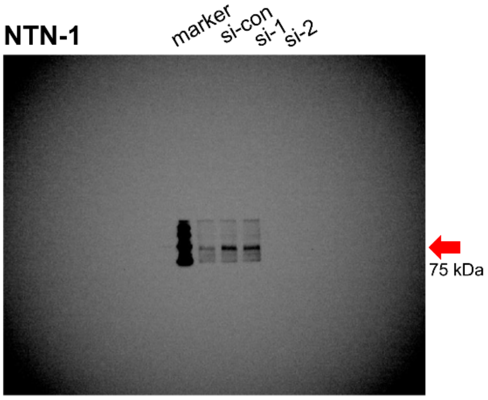

Marker of protein standard from top to bottom:
220, 120, 100, 80 kDa

BAD

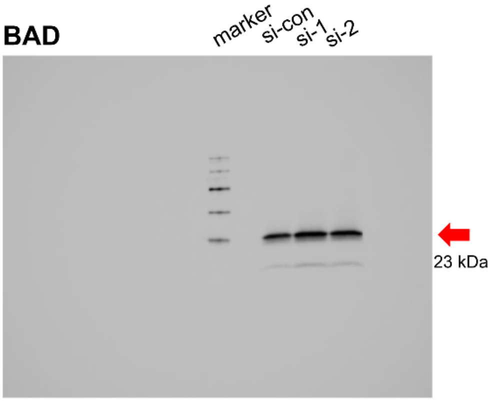

Marker of protein standard from top to bottom
60, 50, 40, 30, 20 kDa

MET

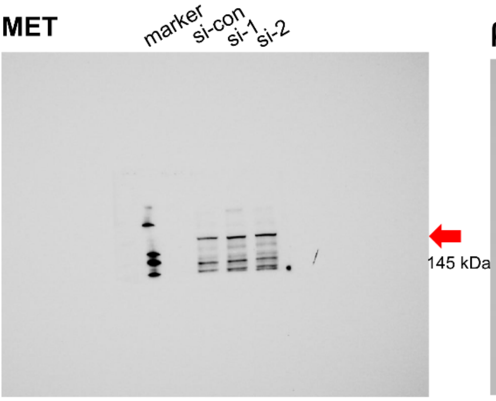

Marker of protein standard from top to bottom:
220, 120, 100, 80 kDa

β-actin

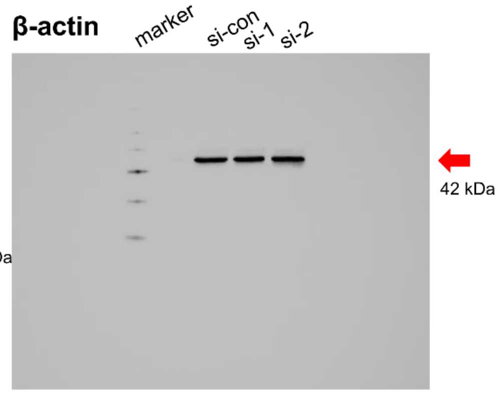

Marker of protein standard from top to bottom:
60, 50, 40, 30, 20 kDa

Supplementary Figure S3. Uncropped gel image for Figure 4

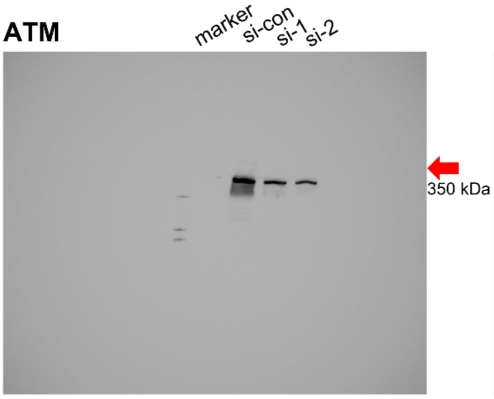

Marker of protein standard from top to bottom
220, 120, 100, 80 kDa

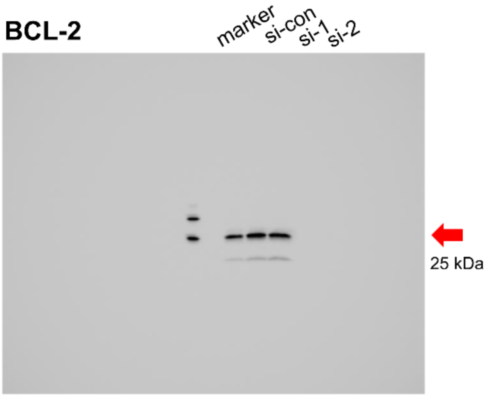

Marker of protein standard from top to bottom
30, 20 kDa

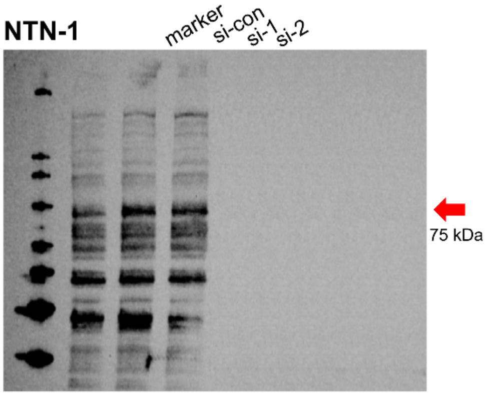

Marker of protein standard from top to bottom
220, 120, 100, 80 kDa

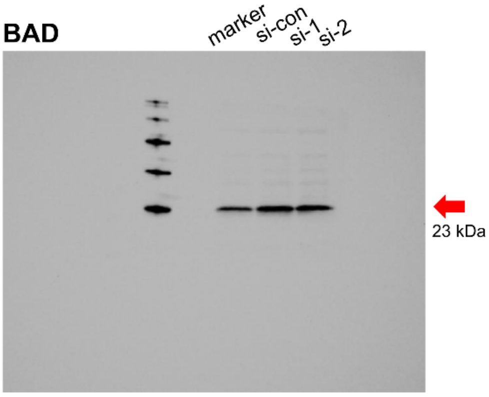

Marker of protein standard from top to bottom:
60, 50, 40, 30, 20 kDa

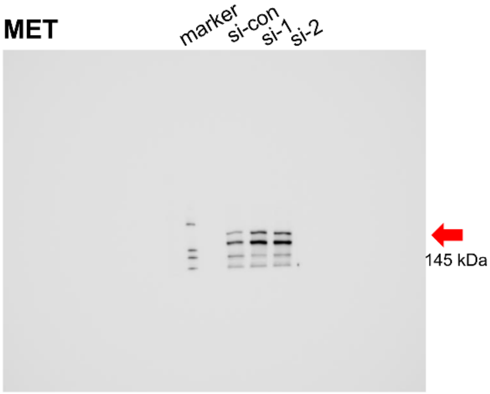

Marker of protein standard from top to bottom:
220, 120, 100, 80 kDa

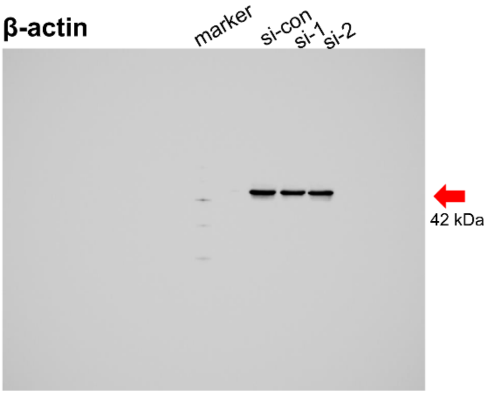

Marker of protein standard from top to bottom:
60, 50, 40, 30, 20 kDa

Supplementary Figure S4. Uncropped gel image for Figure 4

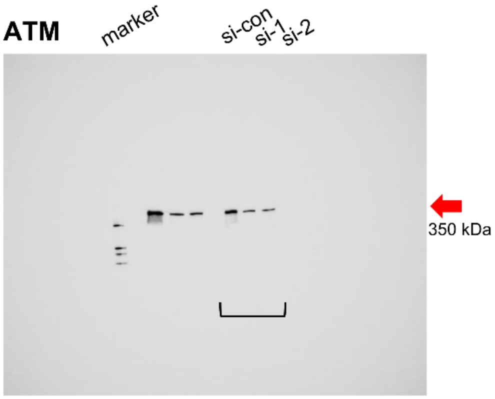

Marker of protein standard from top to bottom
220, 120, 100, 80 kDa

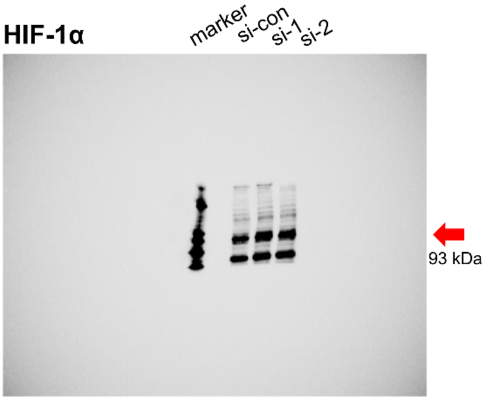

Marker of protein standard from top to bottom:
220, 120, 100, 80 kDa

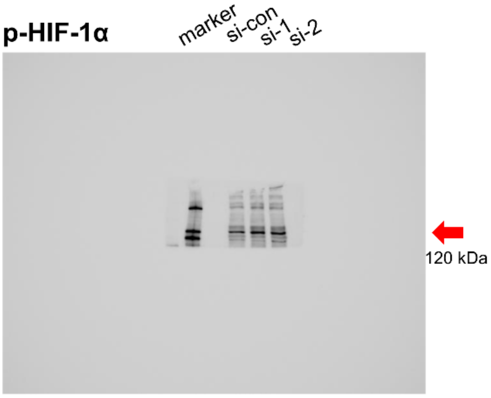

Marker of protein standard from top to bottom:
220, 120, 100, 80 kDa

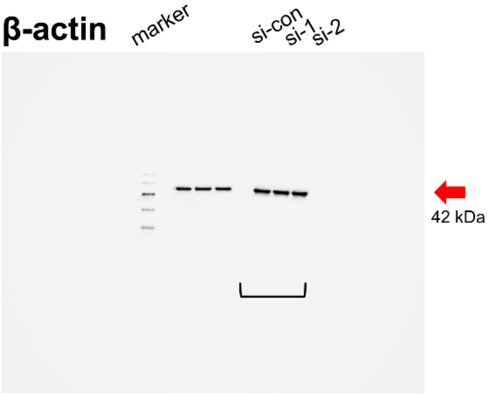

Marker of protein standard from top to bottom:
60, 50, 40, 30, 20 kDa

Supplementary Figure S5. Uncropped gel image for Figure 5

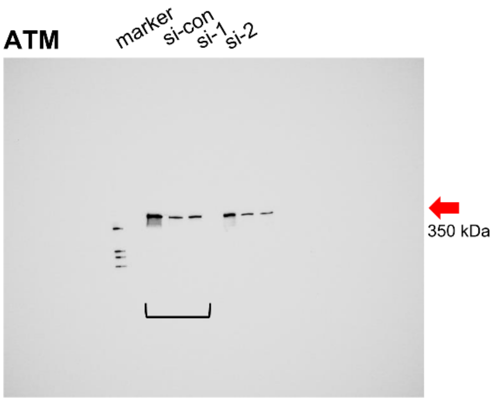

Marker of protein standard from top to bottom:
220, 120, 100, 80 kDa

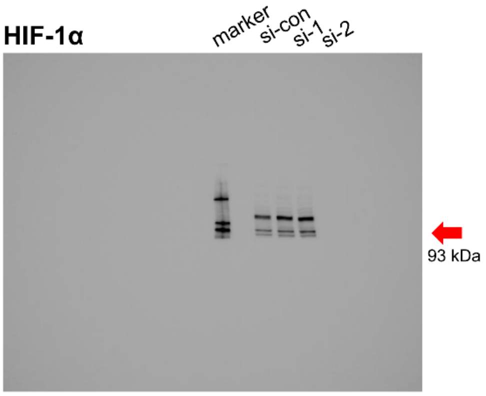

Marker of protein standard from top to bottom:
220, 120, 100, 80 kDa

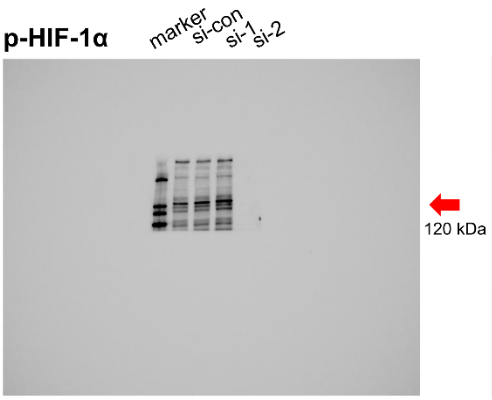

Marker of protein standard from top to bottom:
220, 120, 100, 80 kDa

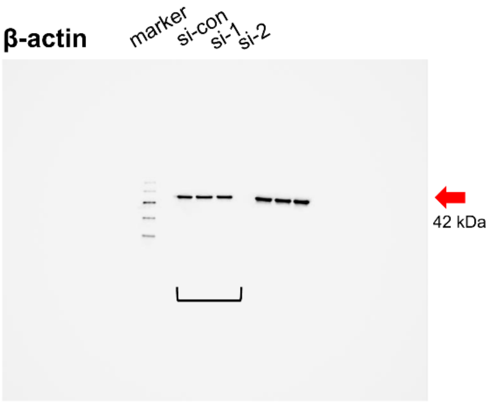

Marker of protein standard from top to bottom:
60, 50, 40, 30, 20 kDa

Supplementary Figure S6. Uncropped gel image for Figure 5
